# RNA-Seq and iTRAQ reveal multiple pathways involved in storage root formation and development in sweet potato (*Ipomoea batatas* L.)

**DOI:** 10.1186/s12870-019-1731-0

**Published:** 2019-04-11

**Authors:** Tingting Dong, Mingku Zhu, Jiawen Yu, Rongpeng Han, Cheng Tang, Tao Xu, Jingran Liu, Zongyun Li

**Affiliations:** Jiangsu Key Laboratory of Phylogenomics & Comparative Genomics, School of Life Science, Jiangsu Normal University, Xuzhou, Jiangsu Province People’s Republic of China

**Keywords:** Sweet potato, Storage root, Transcriptome, iTRAQ, Starch, Plant hormone

## Abstract

**Background:**

Sweet potato (*Ipomoea batatas* L.) is the sixth most important food crop in the world. The formation and development of storage roots in sweet potato is a highly complicated and genetically programmed process. However, the underlying mechanisms of storage root development have not yet been elucidated.

**Results:**

To better understand the molecular mechanisms involved in storage root development, a combined analysis of the transcriptome and proteome of sweet potato fibrous roots (F) and storage roots at four different stages (D1, D3, D5 and D10) was performed in the present study. A total of 26,273 differentially expressed genes were identified in a comparison between the fibrous root library and four storage root libraries, while 2558 proteins showed a 1.0-fold or greater expression difference as indicated by isobaric tags for relative and absolute quantitation (iTRAQ) analysis. The combination of the transcriptome and proteome analyses and morphological and physiological data revealed several critical pathways involved in storage root formation and development. First, genes/proteins involved in the development of meristems/cambia and starch biosynthesis were all significantly upregulated in storage roots compared with fibrous roots. Second, multiple phytohormones and the genes related to their biosynthesis showed differential expression between fibrous roots and storage roots. Third, a large number of transcription factors were differentially expressed during storage root initiation and development, which suggests the importance of transcription factor regulation in the development of storage roots. Fourth, inconsistent gene expression was found between the transcriptome and proteome data, which indicated posttranscriptional regulatory activity during the development of storage roots.

**Conclusion:**

Overall, these results reveal multiple events associated with storage root development and provide new insights into the molecular mechanisms underlying the regulatory networks involved in storage root development.

**Electronic supplementary material:**

The online version of this article (10.1186/s12870-019-1731-0) contains supplementary material, which is available to authorized users.

## Background

Sweet potato (*Ipomoea batatas* L.) is the sixth most important food crop in the world and is widely grown globally due to its high and stable yield, ease of management, high nutrient content and multiple uses [1, 2]. Storage roots constitute the most economically important agronomic trait in sweet potato production, and clarifying the mechanisms underlying storage root development is important for improving the sweet potato yield.

The formation and development of storage roots involve a complex biological process that is regulated by both internal and external factors. At the anatomical level, the storage root of sweet potato is a type of abnormal root that develops from adventitious roots [3], and the formation of storage roots involves the genesis and development of the primary and secondary vascular cambium and several anomalous meristems [4]. After this development, the storage roots exhibit continuous expansion, and large amounts of photosynthates, particularly starch, fill in the parenchyma cells of the developed roots [5].

The initiation and development of storage roots are closely related to the content of endogenous phytohormones. Storage root bulking is the result of the synergistic action of various endogenous plant hormones, such as cytokinins (CTKs), abscisic acid (ABA), auxin (IAA), jasmonic acid (JA) and gibberellin (GAs) [4]. A previous study suggested that the content of trans-zeatin riboside (t-ZR) increases rapidly during the early stage of storage root bulking, and is 6–7 fold greater in storage roots than in fibrous roots [6]. Moreover, exogenous applications of CTKs have been proven to subserve storage root initiation [7, 8]. Thus, CTKs might play a key role in the initiation and the expansion rate of storage roots. Wang et al. [9] reported that the content of endogenous ABA in the storage roots of *Ipomoea batatas* is notably higher than that in the non-storage roots of *Ipomoea trifida*. Furthermore, Nakatani and Komeichi [10] suggested that the ABA levels in the vascular cambium zone are considerably higher than those in other zones, including the xylem, phloem and peripheral zones. The endogenous IAA level increases gradually during the early stage of storage root formation and then decreases in the late stage of storage root formation [11]. Moreover, Ravi et al. [12] reported that a high IAA level is conducive to the promotion of cell division and storage root development. Additionally, JA and GAs reportedly play important roles in the development of storage roots. Specifically, JA is found at high and low levels in the storage and non-storage roots of *Ipomoea trifida*, respectively [7], and the exogenous application of JA promotes an increase in storage root diameter by inducing the expansion of cortex cells [13]. The content of GA4 in storage roots is decreased at the early stage, increased at the middle stage, and decreased again at the late stage [9].

Due to the development of sequencing technologies, the whole genomes of the *Ipomoea trifida* and *Ipomoea batatas* have been successively completed and various genes related to storage root development have been cloned. We grouped these genes into four categories: (1) Cell division- and meristem development-related genes. For example, the expression of the cell division-regulator genes *Cyclin A-like* and *Cyclin D-like* in storage roots at the initial stage is significantly higher than that in the fibrous roots [5]. (2) Expansion-related protein-coding genes. Noh et al. [14] reported that *IbEXP1* inhibits the formation of storage roots, and the silencing of this gene leads to the early development of storage roots and significantly increases the number and total weight of storage roots. (3) Lignin synthesis-related genes. Lignification of the middle column inhibits the conversion of adventitious roots to storage roots [15]. RNA-seq data have shown that genes involved in lignin synthesis, such as the *coumaroyl-CoA synthase gene*, *caffeoyl-CoA O-methyltransferase gene* and *cinnamyl alcohol dehydrogenase gene*, are expressed at higher levels in storage roots at the initial stage than in fibrous roots [5]. (4) Transcription factors. Recent studies have revealed that the MADS-box protein, which plays an important regulatory role in plant growth and development, is an essential regulator in the formation and development of storage roots. For example, Kim et al. [16, 17] cloned five MADS-box genesthat are abundantly expressed during the formation and development of storage roots, i.e., *IbMADS3*, *IbMADS4*, *IbMADS79*, *IbAGL17* and *IbAGL20*. Ku et al. [18] reported that *IbMADS1* regulates the enlargement of the lateral roots of potato. The results reported by Noh showed that *SRD1* influences the formation and development of sweet potato storage roots by regulating the synthesis of auxin [11]. In addition, the homeobox and NAC genes are important regulators of storage roots during the development. Three KNOXI homeobox genes, *Ibkn1*, *Ibkn2* and *Ibkn3* are abundantly found to be expressed at the initial stage [5, 6], and two *NAC* genes are downregulated in storage roots [19].

Understanding the physiology and molecular mechanisms of storage root formation and development is important for improving the yield and quality of sweet potato. Although several studies have investigated the mechanisms underlying storage root development, the relevance of the identified genes related to storage root initiation and development has only been proven at the transcriptional level, and the relationships and interactions among morphological, physiological and genetic changes during the process of storage root development have not yet been elucidated. Here, anatomical, physiological, transcriptome and proteome analyses of fibrous roots and storage roots at different developmental stages were performed. These analyses showed that specific genes and proteins associated with starch and phytohormone synthesis as well as various transcription factors are involved in storage root formation and development and indicate that the formation and development of storage roots constitute a highly complicated and genetically programmed process that requires the participation of multiple regulators.

## Results

### Morphology and anatomy of fibrous roots and storage roots at different stages

**Fig. 1:**
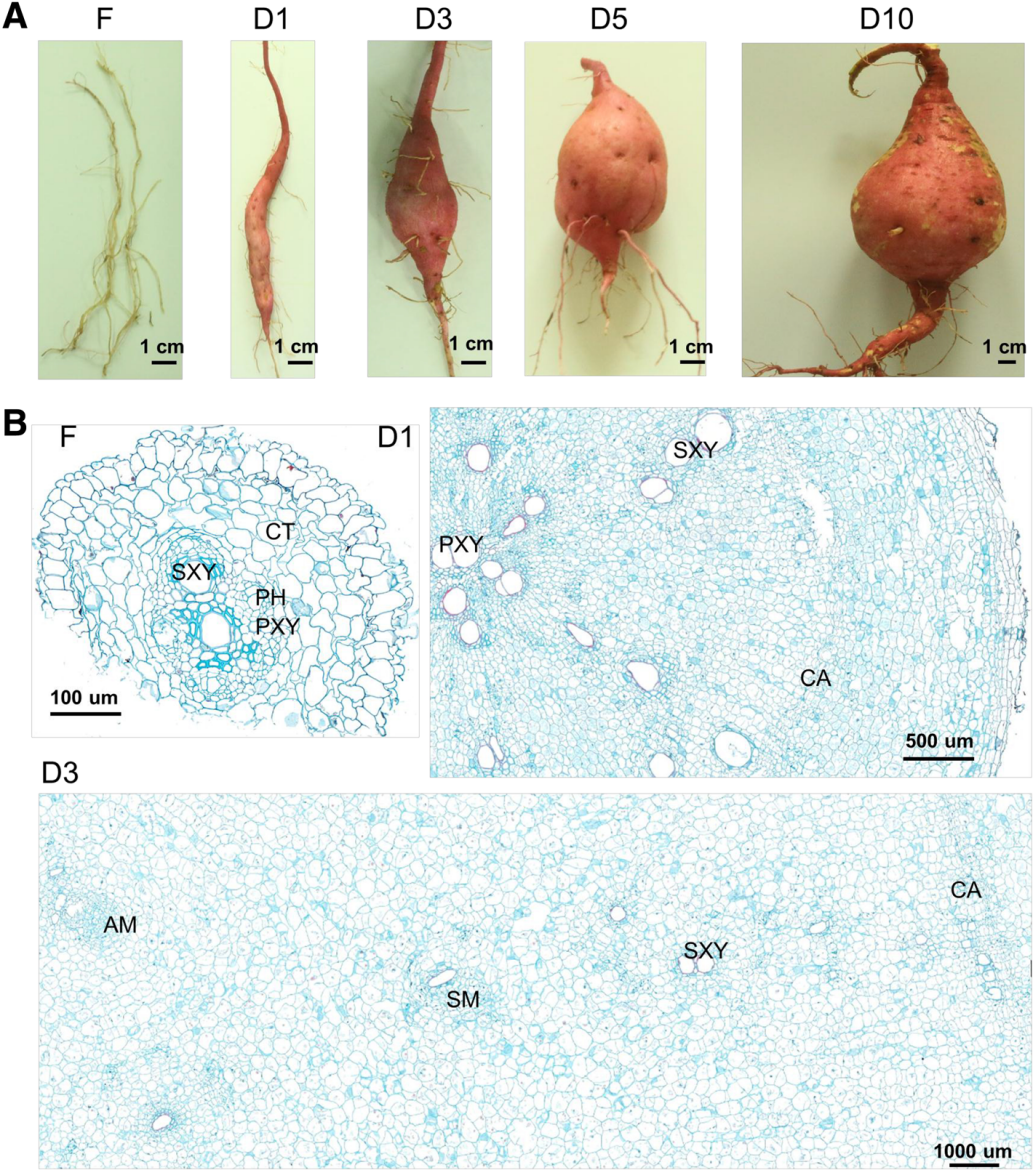
Morphology and anatomy of fibrous roots and sweet potato storage roots at different stages. **a** Phenotypic characterization of fibrous roots and storage roots at different stages. **b** Transverse sections of fibrous roots and storage roots at different stages. F, fibrous root (diameter of approximately 1 mm); D1, initial storage root (diameter of approximately 1 cm); D3, storage root (diameter of approximately 3 cm); D5, storage root (diameter of approximately 5 cm); D10, storage root (diameter of approximately 10 cm); CT, cortex; PH, phloem; PXY, protoxylem; SXY, secondary xylem; CA, cambium; SM, secondary meristem; AM, anomalous meristem

To elucidate the mechanism of storage root initiation and development, sweet potato roots at five distinct stages of development were collected for measurement of the maximal root diameter (Fig. 1a). The first step of the process of storage root formation involves the formation of adventitious roots (root diameter: 1 mm, F), and as the development process continues, some adventitious roots begin thickening and form pencil roots (diameter: 1 cm, D1). The pencil roots then gradually develop into storage roots (diameter: 3 cm, D3; diameter: 5 cm, D5; diameter: 10 cm, D10). To cover the whole root development process, sweet potato roots were collected at 15, 30, 60, 90 and 120 days after transplanting, which correspond to the five above-described stages of root formation.

Moreover, the root anatomical observations of fibrous roots and D1- and D3-stage storage roots were performed. As shown in Fig. 1b, the fibrous roots (F) displayed small steles and large cortices, which are typical anatomical characteristics of normal young roots. An obvious circular primary cambium in the initial storage roots (D1), and a secondary cambium and anomalous meristems were later observed in the storage roots at later stages (D3). These results indicated that meristem activity is the main driver of storage root formation and development.

### Transcriptome analysis of fibrous roots and storage roots at different stages

To investigate the molecular mechanisms underlying the formation and development of sweet potato storage roots, five cDNA libraries were prepared from the middle section of fibrous roots (F) and storage roots at four different stages (D1, D3, D5 and D10), and subjected to RNA-Seq analysis using the Illumina HiSeq 2000 platform. After the removal of adaptors and reads containing unknown or low-quality nucleotides, more than 20,000,000 clean reads were obtained from the fibrous root (F) and the D1, D3, D5 and D10 storage root libraries (Table 1), and these reads then were aligned to the sweet potato genome (http://public-genomes-ngs.molgen.mpg.de/cgi-bin/hgGateway?hgsid=9052&clade=plant&org=Ipomoea+batatas&db=ipoBat4) using HISAT [20]. As shown in Table 1, more than 69% of the total reads were mapped to the genome, and less than 5% of the reads mapped to multiple sites. Moreover, to acquire the most informative and complete annotation, all the sequences were matched against the NR, NT, GO, KO, KOG, Pfam and SwissProt databases by BLASTX (e ≤ 1.00 × 10^−^ 5). Of all the transcripts, 76.17% (86, 743) were aligned against the NR database, 49.69% (56, 585) transcripts were aligned against the NT databases, and 34,938 (30.68%), 65,625 (57.63%), 58,452 (51.33%), 58,923 (51.74%), and 20,565 (18.06%) showed significant similarity to known gene/proteins in the KO, SwissProt, Pfam, GO and KOG databases, respectively (Additional file 1: Figure S1).

**Table 1:** Summary of the transcriptome data in sweet potato roots

sample	Raw reads	Clean reads	Clean bases	Total map	Unique map	Multi map	Splice map
F_1	26,124,764	25,442,025	7.63G	69.24%	65.01%	4.23%	23.53%
F_2	25,889,827	25,359,234	7.61G	69.93%	65.74%	4.19%	23.83%
F_3	25,876,842	25,016,010	7.5G	71.83%	67.42%	4.41%	24.55%
D1_1	22,551,090	21,935,933	6.58G	75.58%	71.04%	4.54%	24.38%
D1_2	28,567,062	28,008,903	8.4G	75.0%	70.35%	4.65%	26.3%
D1_3	26,290,672	25,685,971	7.71G	74.25%	70.05%	4.2%	23.12%
D3_1	24,556,185	23,919,071	7.18G	75.33%	70.85%	4.49%	26.12%
D3_2	25,349,499	24,676,501	7.4G	77.25%	72.28%	4.97%	25.97%
D3_3	25,184,630	24,501,723	7.35G	75.68%	70.92%	4.77%	25.59%
D5_1	28,616,823	27,935,363	8.38G	75.74%	71.38%	4.35%	25.03%
D5_2	26,397,886	25,447,938	7.63G	77.42%	72.16%	5.26%	23.36%
D5_3	23,103,960	22,311,013	6.69G	77.14%	71.65%	5.49%	21.79%
D10_1	25,648,113	25,081,452	7.52G	76.69%	72.1%	4.59%	26.4%
D10_2	27,226,706	26,638,262	7.99G	76.58%	71.78%	4.8%	24.14%
D10_3	25,359,719	24,689,508	7.41G	75.29%	70.95%	4.34%	23.74%

**Fig. 2:**
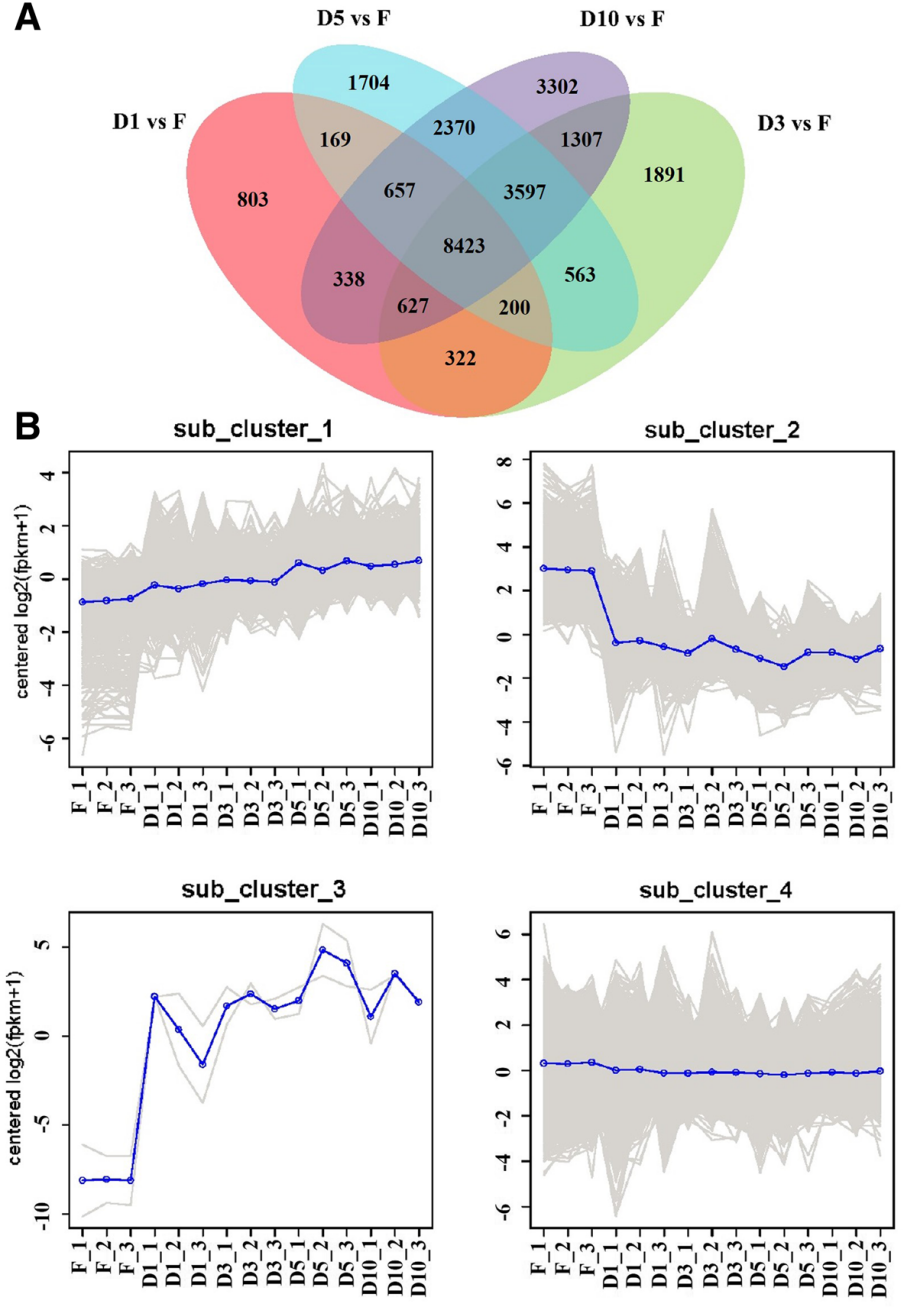
Differentially expressed genes between storage roots and fibrous roots of sweet potato in transcriptome. **a** Number of differentially expressed transcripts between D1 stage sorage roots and fibrous roots (D1 vs F), D3 stage sorage roots and fibrous roots (D3 vs F), D5 stage sorage roots and fibrous roots (D3 vs F) as well as D10 stage sorage roots and fibrous roots (D10 vs F). **b** Clusters of differentially expressed genes. 1359 DEGs are contained in sub_cluster_1, 1699 DEGs are contained in sub_cluster_2, 2 DEGs are contained in sub_cluster_3, and 23,366 DEGs are contained in sub_cluster_4. F_1, F_2 and F_3, fibrous root; D1_1, D1_2 and D1_3, initial storage root (diameter is about 1 cm); D3_1, D3_2 and D3_3, storage root (diameter is about 3 cm); D5_1, D5_2 and D5_3, storage root (diameter is about 5 cm); D10_1, D10_2 and D10_3, storage root (diameter is about 10 cm)

To profile gene expression, the expression levels of genes were measured and analyzed. Based on the false discovery rate (FDR) ≤ 0.05, and fold change (FC) ≥ 2, a total of 26,273 DEGs were identified from the fibrous root library compared with the four storage root libraries. Of these, 11,539 DEGs were from the fibrous root library compared with the D1 storage root library, 16,930 DEGs were from the fibrous root library compared with the D3 storage root library, 17,683 DEGs were from the fibrous root library compared with the D5 storage root library, and 20,621 DEGs were from the fibrous root library compared with the D10 storage root library (Fig. 2a and Additional file 2: Table S1).

To cluster the genes showing similar expression profiles during the formation and development of storage roots, hierarchical clustering analysis was performed. Four prominent gene subclusters were identified (Fig. 2b and Additional file 3: Table S2). The genes in subcluster 1 were gradually upregulated starting at the D1 stage (Fig. 2b), whereas the genes in subcluster 4 showed no significant difference among the four stages of storage root development (Fig. 2b). In contrast, the genes in subcluster 2 were strongly downregulated from the F to the D1 stage and then showed slightly decreases from the D1 to D10 stage (Fig. 2b). Conversely, the genes in subcluster 3 were strongly upregulated from the D1 to D10 stage (Fig. 2b). Among the identified DEGs, 4.0% showed changes in abundance of more than 5-fold, and the highest expression change was 23-fold (Additional file 3: Table S2). A GO analysis of the subclustered genes revealed the enrichment of the genes at different stages of storage root formation and development (Additional file 4: Figure S2 and Additional file 5: Table S3). The genes in subcluster 1 were found to be involved in carbohydrate metabolic processes. For example, sucrose synthase (novel.75288) and starch phosphorylase (novel.51674) and xylosyltransferase (Tai6.43252) formed part of this subcluster, and hormone-related genes, such as ethylene-responsive transcription factor (Tai6.15383), auxilin-related protein (Tai6.31273), and transcription factors, including MADS-box23 (Tai6.15221), bZIP44 (Tai6.1410) and BEL1 (Tai6.36202), were also found in this subcluster. Two genes in subcluster 3, 1,4-alpha-D-glucan maltohydrolase (Tai6.6953) and sporamin B (novel.15262), were identified (Additional file 3: Table S2).

### Proteomic analysis and transcriptome-proteome matching

In parallel, a comparative proteome analysis was performed on the fibrous roots and storage roots at different stages by the iTRAQ. A total of 7727 proteins were identified in the five libraries. Of these proteins, 98.81% (7635) were annotated in the search against the GO database, and 88.08% (6806) transcripts were aligned against the IPR databases. In addition, 6715 (87.03%) and 4373 (56.59%) showed significant similarity to known proteins in the KEGG and COG databases, respectively (Additional file 6: Figure S3).

**Fig. 3:**
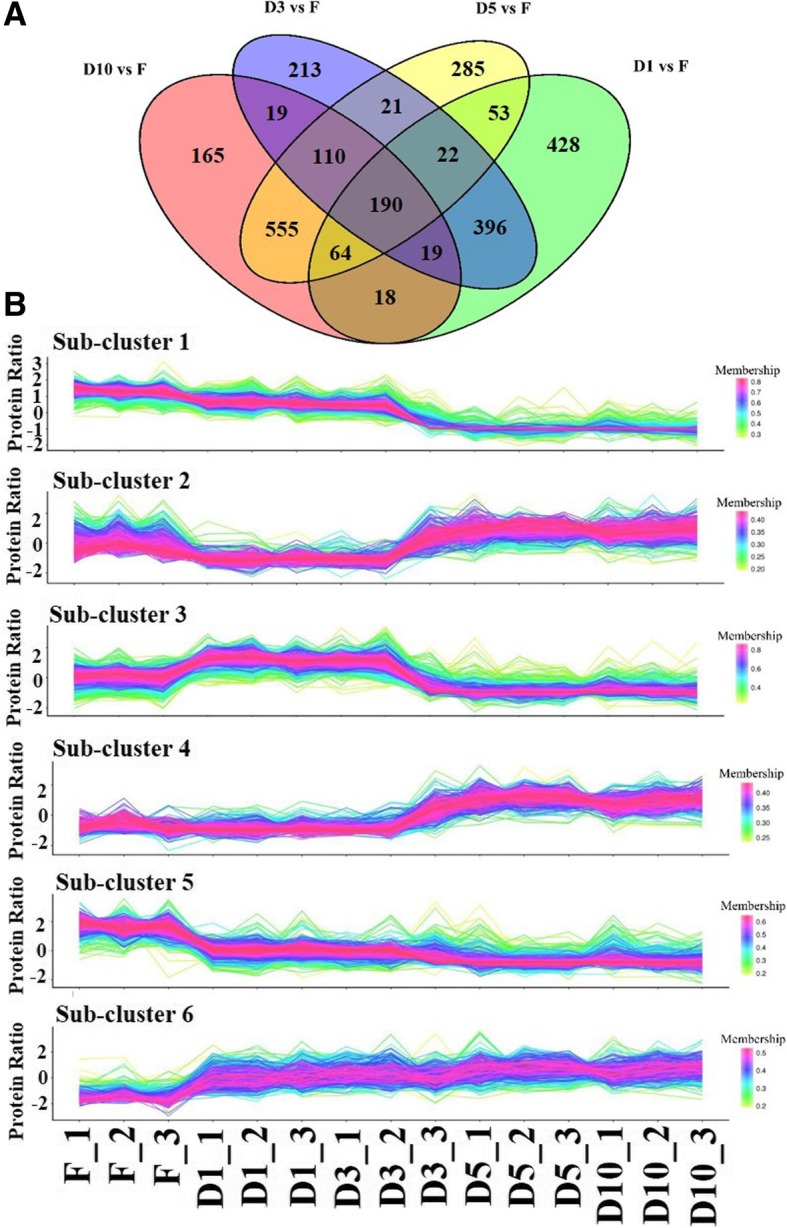
Differential proteins between storage roots and fibrous roots of sweet potato in proteome. **a** Number of differential proteins between D1 stage sorage roots and fibrous roots (D1 vs F), D3 stage sorage roots and fibrous roots (D3 vs F), D5 stage sorage roots and fibrous roots (D5 vs F) as well as D10 stage sorage roots and fibrous roots (D10 vs F). **b** Clusters of differential proteins. F_1, F_2 and F_3, fibrous root; D1_1, D1_2 and D1_3, initial storage root (diameter is about 1 cm); D3_1, D3_2 and D3_3, storage root (diameter is about 3 cm); D5_1, D5_2 and D5_3, storage root (diameter is about 5 cm); D10_1, D10_2 and D10_3, storage root (diameter is about 10 cm). Coloring correlates with the membership value, the higher the membership value, the closer the expression pattern among the protein members

The comparison of the fibrous root (F) library with the four storage root libraries (D1, D3, D5 and D10) identified 2558 DEPs, and 1190, 990, 1300 and 1140 of these DEPs were obtained from the comparisons of the F root library with the D1, D3, D5, and D10 storage root libraries, respectively (Fig. 3a and Additional file 7: Table S4).

A hierarchical clustering analysis of the DEPs revealed six prominent protein clusters (Fig. 3b). Subcluster 1 showed intense downregulation from the D3 to D5 stage (Fig. 3b). Conversely, subcluster 4 was strongly upregulated from the D3 to D5 stage (Fig. 3b). The proteins in subcluster two were inhibited at the D1 and D3 stages and returned to their baseline levels at the D5 stage (Fig. 3b), whereas the opposite trend was obtained for the proteins in subcluster 3 (Fig. 3b). Additionally, subcluster 6 was significantly upregulated from the F to D1 stage and then slightly increased from the D1 to D10 stage, whereas the opposite trend was found for subcluster 5 (Fig. 3b). A GO analysis of the clustered genes revealed that the most abundant terms in subclusters 3 and 6 were “binding” under the molecular function category and “metabolic process” under the biological process category, respectively (Additional file 8: Figure S4 and Additional file 9: Table S5).

**Fig. 4:**
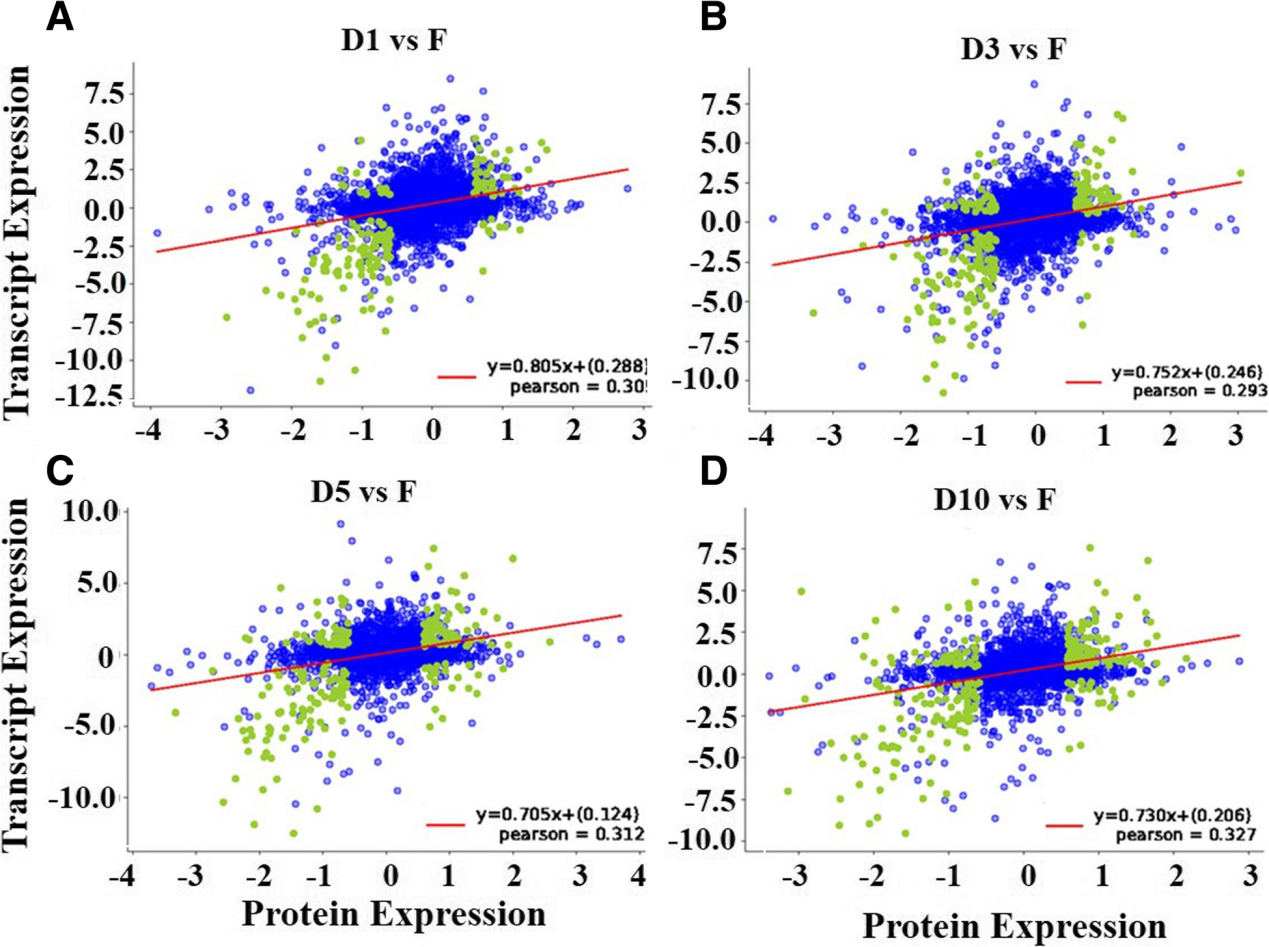
The Pearson correlation coefficient of transcriptome and proteome between storage roots and fibrous roots. **a** The Pearson correlation coefficient of transcriptome and proteome between D1 stage storage roots and fibrous roots. **b** The Pearson correlation coefficient of transcriptome and proteome between D3 stage storage roots and fibrous roots. **c** The Pearson correlation coefficient of transcriptome and proteome between D5 stage storage roots and fibrous roots. **d** The Pearson correlation coefficient of transcriptome and proteome between D10 stage storage roots and fibrous roots. Green and blue dots represent the differential and consistent gene/protein expression, respectively. The expression data were clustered according to the Log2-transformed values

Because the proteome data were obtained from the same root samples used to produce the transcriptome data, we matched the identified proteins with transcripts from the RNA-Seq data. Of the 7727 identified proteins, 4110 had corresponding transcripts in the transcriptome data (Additional file 7: Table S4). A linear regression analysis revealed that the Pearson correlation coefficient was approximately 0.3 (Fig. 4), which indicates a weak correlation between the transcriptome and the proteome profiles. As shown in Additional file 10: Figure S5A, the transcript levels of 158 out of 1098 DEPs showed significant differences in the comparison of the fibrous roots with the D1-stage storage roots. The comparison of the fibrous roots with the D3-stage storage roots showed that 207 out of 926 DEPs exhibited significant transcript-level differences (Additional file 10: Figure S5B). A total of 227 out of 1225 DEPs showed significant differences in transcript levels in the fibrous roots compared with the D5-stage storage roots (Additional file 10: Figure S5C). Additionally, the comparison of the fibrous roots with the D10-stage storage roots showed that 258 out of 1074 DEPs exhibited significant transcript-level differences (Additional file 10: Figure S5D). Similarly to many previously reported studies [21–23], these results revealed that not all the mRNA: protein ratios reflected significant changes at both the transcript and protein levels. This finding indicates either a technical limitation of the proteome approach that makes comparisons with transcriptome data difficult, or the occurrence of posttranscriptional regulation during the development of storage roots.

### Starch accumulates during storage root formation and development

**Fig. 5:**
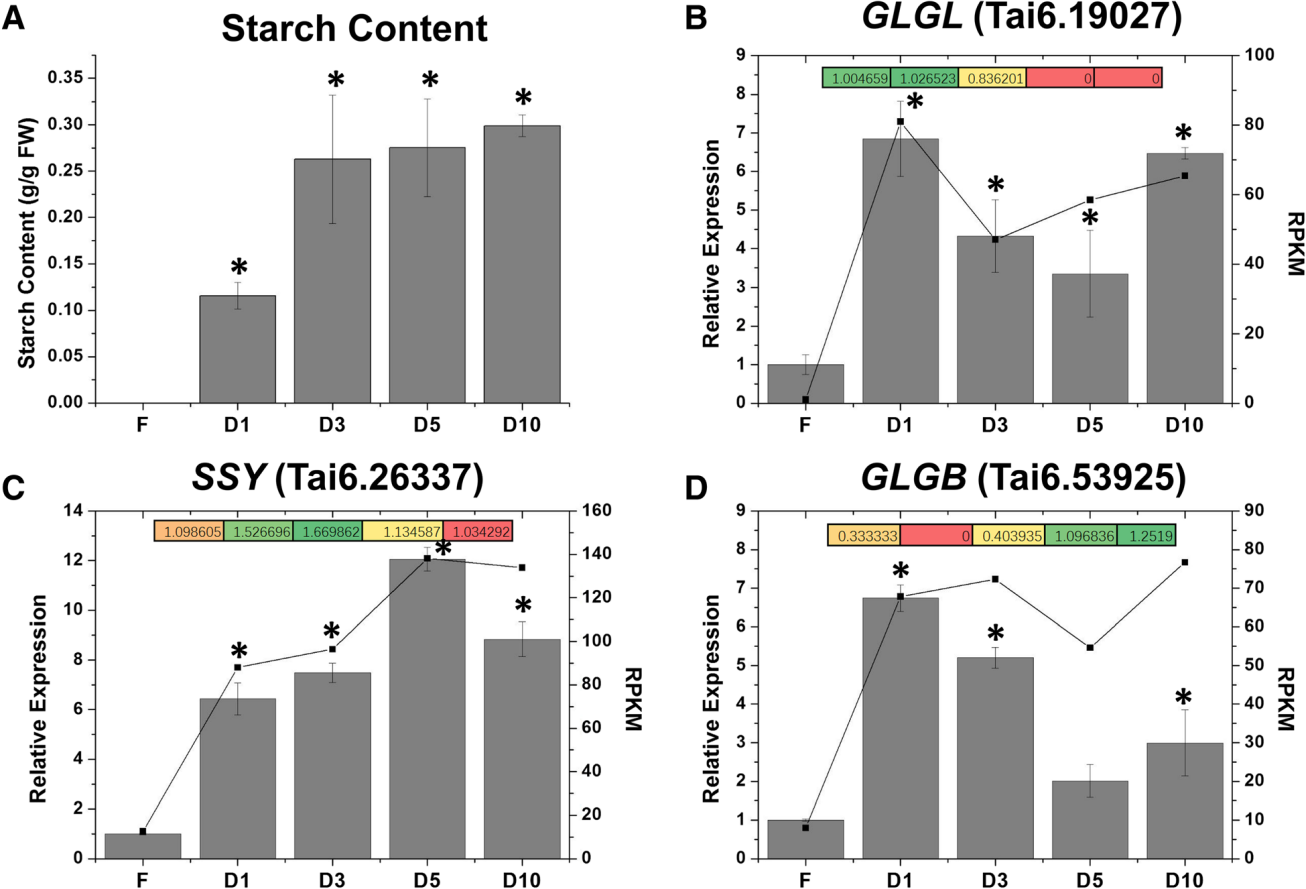
Trends in starch content and starch biosynthetic enzymes/genes during storage root development. **a** The content of starch during storage root development. **b** The trend of *GLGL* (*ADP-glucose pyrophosphorylase gene*, Tai6. 19,027) expression in transcriptome (black points and lines) and proteome (colored boxes), as well as its qRT-PCR validation (grey bars). **c** The trend of *SSY* (*starch synthase gene*, Tai6. 26,337) expression in transcriptome (black points and lines) and proteome (colored boxes), as well as its qRT-PCR validation (grey bars). **d** The trend of *GLGB* (*starch-branching enzyme gene*, Tai6. 53,925) expression in transcriptome (black points and lines) and proteome (colored boxes), as well as its qRT-PCR validation (grey bars). FPKM (fragments per kolibase of transcript per million fragments mapped) values were used to represent the relative expression of genes in transcriptome. FC (Fold Change) values were used to represent the relative expression of proteins in proteome

Starch is the most important dry matter in sweet potato. To clarify the dynamic changes in starch accumulation in sweet potato, the starch contents in fibrous roots and storage roots at different stages were detected. The results suggested that nearly no starch accumulated in fibrous roots (Fig. 5a). The storage roots started to show rapid starch accumulation at the initial stage, and at later stages during root development, the starch content in storage roots increased gradually and reached 0.3 g/g fresh weight at the D10 stage (Fig. 5a).

To explore the molecular mechanism underlying starch accumulation during the development of storage roots, several starch synthesis-related enzymes were identified. The RNA-Seq data showed that most of the starch biosynthesis-related genes were highly upregulated in storage roots compared with fibrous roots (Additional file 11: Table S6). Consistent with the RNA-Seq data, the proteome data also suggested that most of the starch biosynthesis-related proteins were upregulated in storage roots compared with fibrous roots (Additional file 11: Table S6). To further confirm these results, the transcript levels of these starch biosynthesis-related enzymes in fibrous roots and storage roots were examined by qRT-PCR. The results showed that starch biosynthesis-related genes, including *GLGL* (Tai6.19027), *SSY* (Tai6.26337) and *GLGB* (Tai6.53925), were all highly upregulated in the storage roots (Fig. 5 and Additional file 12: Figure S6). These results were consistent with those obtained in the transcriptome analysis and further confirmed the reliability of the RNA-Seq and proteome analyses.

### Accumulation of plant hormones during storage root formation and development

**Fig. 6:**
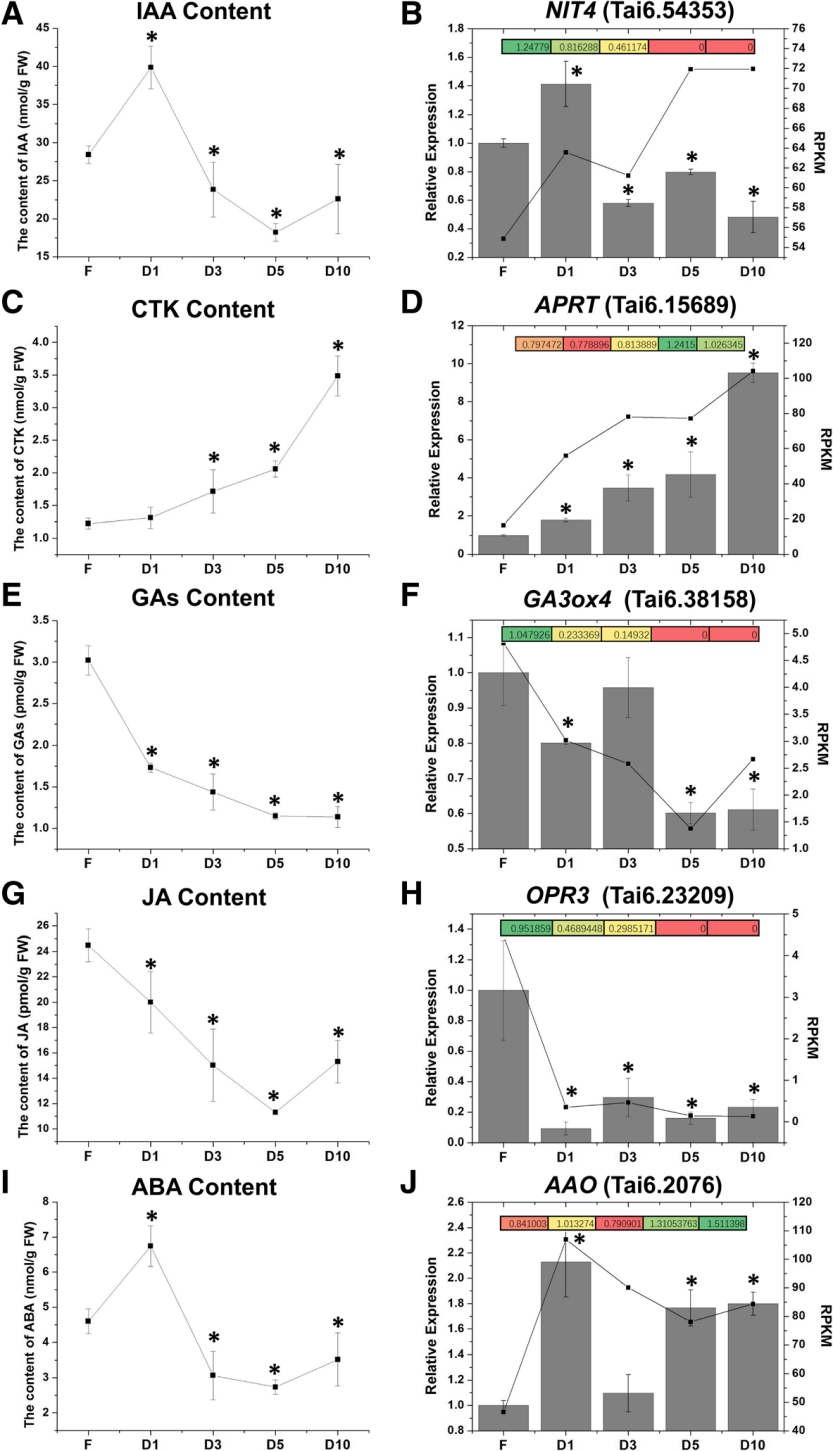
Trends in endogenous phytohormone contents and their biosynthetic enzymes/genes during storage root development. **a** The content of IAA during storage root development. **b** The trend of *NIT*4 (*nitrile aminohydrolase 4 gene*, Tai6. 54,353) expression in transcriptome (black points and lines) and proteome (colored boxes), as well as its qRT-PCR validation (grey bars). **c** The content of CTK during storage root development. **d** The trend of *APRT* (*adenine hosphoribosyl transferase gene*, Tai6. 15,689) expression in transcriptome (black points and lines) and proteome (colored boxes), as well as its qRT-PCR validation (grey bars). **e** The content of GAs during storage root development. **f** The trend of *GA3ox4* (*gibberellin 3-β-dioxygenase 4 gene*, Tai6. 38,158) expression in transcriptome (black points and lines) and proteome (colored boxes), as well as its qRT-PCR validation (grey bars). **g** The content of JA during storage root development. **h** The trend of *OPR3* (*OPDA reductase 3 gene*, Tai6. 23,209) expression in transcriptome (black points and lines) and proteome (colored boxes), as well as its qRT-PCR validation (grey bars). **i** The content of ABA during storage root development. **j** The trend of *AAO* (*ABA-aldehyde oxidase gene*, Tai6. 2076) expression in transcriptome (black points and lines) and proteome (colored boxes), as well as its qRT-PCR validation (grey bars). FPKM (fragments per kolibase of transcript per million fragments mapped) values were used to represent the relative expression of genes in transcriptome. FC (fold change) values were used to represent the relative expression of proteins in proteome

Previous reports have suggested a close relationship between storage root development and plant hormones. Thus, the contents of several related phytohormones including indole acetic acid (IAA), cytokinins (CTKs), gibberellins (GAs), jasmonic acid (JAs) and abscisic acid (ABA) in sweet potato fibrous roots and storage roots were detected. As shown in Fig. 6a and i, the levels of IAA and ABA were significantly induced in the storage roots at the initial stage compared with the fibrous roots, and after this induction, the levels declined gradually until reaching their lowest values at the D5 stage. The CTK content increased progressively during the root development (from fibrous to D10-stage storage roots) (Fig. 6c). Conversely, the levels of GAs and JAs decreased gradually from the fibrous roots to the storage roots, and the JA content recovered at the D10 stage (Fig. 6e and g).

Additionally, genes/proteins associated with the biosynthesis of these phytohormones were identified from the RNA-seq and proteome data. The RNA-Seq results showed that most of the genes related to the biosynthesis of IAA, CTK and ABA were upregulated in the storage roots compared with the fibrous roots (Additional file 11: Table S6, Fig. 6, and Additional file 12: Figure S6). The expression levels of most genes associated with GA and JA biosynthesis were reduced in the storage roots compared with the fibrous roots (Additional file 11: Table S6 and Fig. 6f and h). The proteome data revealed different results. For example, some proteins, such as NIT4, showed the opposite expression trend compared with those of their corresponding genes (Fig. 6b and h). To further confirm these results, the transcript levels of several of the genes associated with the biosynthesis of these phytohormones in the fibrous and storage roots were examined by qRT-PCR. The results showed that the expression levels of related genes were all consistent with the RNA-Seq data (Fig. 6).

### Diversification of transcription factors during storage root formation and development

**Fig. 7:**
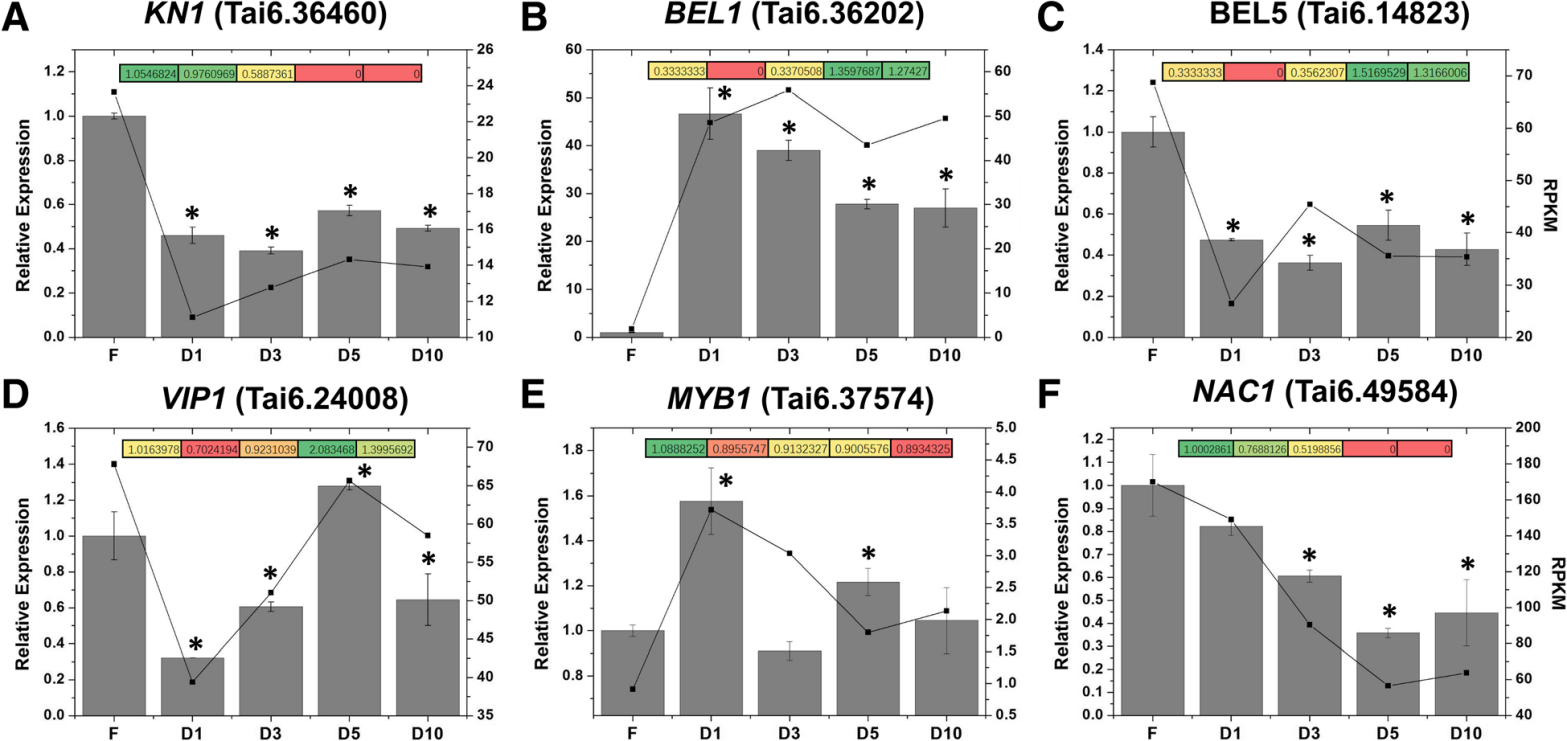
Trends in transcription factors expression during storage root development. **a** The trend of *KN1* (a homeobox family transcription factor, Tai6. 36,460) expression in transcriptome (black points and lines) and proteome (colored boxes), as well as its qRT-PCR validation (grey bars). **b** The trend of *BEL1* (a homeobox family transcription factor, Tai6. 36,202) expression in transcriptome (black points and lines) and proteome (colored boxes), as well as its qRT-PCR validation (grey bars). **c** The trend of *BEL5* (a homeobox family transcription factor, Tai6. 14,823) expression in transcriptome (black points and lines) and proteome (colored boxes), as well as its qRT-PCR validation (grey bars). **d** The trend of *VIP1* (a bZIP family transcription factor, Tai6. 24,008) expression in transcriptome (black points and lines) and proteome (colored boxes), as well as its qRT-PCR validation (grey bars). **e** The trend of *MYB1* (a MYB family transcription factor, Tai6. 37,574) expression in transcriptome (black points and lines) and proteome (colored boxes), as well as its qRT-PCR validation (grey bars). **f** The trend of *NAC1* (a NAC family transcription factor, Tai6. 49,584) expression in transcriptome (black points and lines) and proteome (colored boxes), as well as its qRT-PCR validation (grey bars). FPKM (fragments per kolibase of transcript per million fragments mapped) values were used to represent the relative expression of genes in transcriptome. FC (Fold Change) values were used to represent the relative expression of proteins in proteome

Transcription factors play essential regulatory roles in the initiation and development of storage roots in sweet potato. An analysis of our RNA-Seq data identified more than 3000 differentially expressed transcription factors (Additional file 2: Table S1 and Additional file 13: Table S7), and six of these transcription factors were selected for further investigation. The subsequent analysis suggested that two homeobox genes, i.e., *KN1* and *BEL5*, were significantly downregulated in the storage roots compared with the fibrous roots (Fig. 7a and c), and another homeobox gene, *BEL1*, was substantially upregulated in the storage roots compared with the fibrous roots by more than 40-fold (Fig. 7b). We also found that BEL1 shares only 22.59% similarity with BEL5 at the amino acid level (Additional file 14: Figure S7). The expression of *VIP1*, a bZIP family transcription factor, was strongly reduced in the storage roots at the early stage and then gradually increased until the baseline levels were observed at the D5 stage (Fig. 7d). However, a large number of *MYB1* transcripts were observed in storage roots at the initial stage (Fig. 7e). Moreover, a NAC family gene, *NAC1*, was gradually downregulated in the storage roots compared with the fibrous roots (Fig. 7f). Most of the proteome data and all of the qRT-PCR results were consistent with the RNA-seq data (Fig. 7).

## Discussion

The initiation and bulking of storage roots constitute a highly complicated and genetically programmed process that mainly involves the development of first, second and anomalous cambias and the accumulation of starch and other dry matter [4].

A circular primary vascular cambium forms at the onset of storage root thickening, and several secondary and anomalous cambia are then generated, which leads to the bulking of storage roots and the formation of starch storage tissues [24]. Accordingly, our study showed that the meristems are always active during storage root bulking, which is consistent with the results of previous studies (Fig. 1b). Additionally, the transcriptome data obtained in this study revealed that regulators of meristem development, such as *LBD4* (*LOB domain-containing protein 4*, novel.12886), *WOX4* (*WUSCHEL HOMEOBOX RELATED 14*, Tai6.15498) and *TMO6* (*TARGET OF MONOPTEROS 6*, Tai6.43388), were all substantially upregulated at the early stage of storage root development (Additional file 2: Table S1). Moreover, genes/proteins involved in cell division, including cell division protein FtsZ (Tai6.15786), cell division cycle protein 48 (Tai6.46037) and cell division control protein 2 (Tai6.51075), were also included in the sets of differentially expressed transcripts and proteins (Additional file 7: Table S4). These results indicated that the formation and thickening of storage roots involves active meristems and cell division.

Starch accumulation occurs during the bulking of storage roots. In sweet potato storage roots, starch is mainly synthesized from sucrose that is produced from photosynthesis [25]. Several enzymes including ADP-glucose pyrophosphorylase (GLGL), starch synthase (SSY) and starch-branching enzyme (GLGB), are essential in sweet potato starch biosynthesis [26, 27]. The comparison of storage roots with fibrous roots revealed that starch biosynthesis-related enzymes, including *GLGL* (Tai6.19027), *SSY* (Tai6.26337) and *GLGB* (Tai6.53925), were notably upregulated in the storage roots (Fig. 5). Moreover, notable levels of starch accumulation were detected in storage roots at the early stage (Fig. 5a). These findings indicated that starch starts to accumulate as soon as storage roots begin to expand. Together with the anatomical results, these findings revealed that meristem activity is the driving force of the formation and development of storage roots, and the storage roots then provide places for starch storage. Moreover, all these processes are controlled by endogenous phytohormones, and their expression is controlled by regulatory genes [4].

### Multiple hormones synergistically regulate the development of storage roots

Studies have revealed that the development of storage roots is controlled by endogenous phytohormones [4]. The present study revealed significant accumulation of IAA and ABA in storage roots at the initial stage (Fig. 6a and i). ABA is essential for tuber formation [28], and it might play roles in storage root bulking by activating cell division [9], whereas IAA is considered essential in both the early stage of storage root formation and the thickening of storage roots [10, 11]. Thus, the high accumulations of IAA and ABA detected at the D1 stage might play an important role in the early stages of storage root development. The CTK level increased continuously from the early to late stages of storage root development (Fig. 6c). Previous studies have shown that CTKs are related to both stolon development and tuber initiation [29, 30]. In sweet potato, CTKs appear to be key factor in the formation of storage roots as a prerequirement for cambial cell proliferation [31]. Moreover, the GA and JA contents showed gradual decreases during storage root development (from the fibrous roots to the D-10 stage storage roots) (Fig. 6e and g). The literature shows that JA might induce storage root formation [10], and GAs are thought to play different roles in storage root formation and development [9]. In potato, GAs are important promoters in stolon initiation but serve as inhibitors of tuber initiation [28, 29]. Furthermore, the phytohormone contents were consistent with the expression trends of corresponding biosynthesis-related genes/enzymes (Fig. 6). Thus, we hypothesized that different phytohormones play roles at different stages of storage root development.

Nevertheless, Wang et al. [9] reported that dry storage root yields are positively correlated with the contents of ABA and CTKs, but not with the content of IAA or GA_4_. Furthermore, CTKs cannot trigger storage root formation in the presence of a low sucrose level in the root [32]. Thus, we believe that the formation and development of storage roots are regulated by multiple hormones and other regulators, such as sucrose, in a synergistic manner. Ku et al. [18] reported that ABA stimulates cambial cell division by interacting with CTKs, and this stimulation results in the growth of storage roots. Moreover, in potato, the effects of exogenously applied ABA are dependent on its variety, concentration, and interaction with CTKs [33, 34]. In addition, CTKs and JA exert a synergistic effect on the initiation of storage roots and the transcription levels of storage root development-related genes/regulators, such as IbMADS1, sporamin and IbAGPase [18]. Nevertheless, the synergistic effect of phytohormones such as CTKs, IAA, JAs, ABA and GAs on sweet potato storage root formation and development warrants further investigation.

### Transcription factors play essential roles in the storage root development processes

The developmental program of storage roots, including the formation of cambium meristems, starch biosynthesis and hormone biosynthesis and transport, mostly relies on transcription factor regulation [11, 18]. Our RNA-Seq data identified more than 3000 differentially expressed transcription factors (Additional file 2: Table S1).

Homeobox transcription factors constitute a large family that plays key roles in the development of plants, and recent studies have suggested that these transcription factors play an important role in the regulation of potato tuber expansion. For example, a knotted-like homeobox (KNOX) gene, *POTH1*, regulates the yield of potato tubers by regulating gibberellin synthesis and the CTKs levels [35]. The overexpression of *StBEL5*, a BEL1-like homeobox gene, increases the yield of potato tuber by increasing the CTKs levels [36]. In sweet potato, homeobox genes are reportedly related to storage root development. Previous RNA-Seq data have revealed that three homeobox genes are notably upregulated during the formation and thickening of storage roots [37]. Tanaka et al. [6] reported that three KNOX genes, *Ibkn1*, *Ibkn2* and *Ibkn3*, exhibited high expression in storage roots. The present results showed that at least 15 homeobox genes/proteins were differentially expressed in fibrous roots compared with storage roots (Additional file 11: Table S6). In particular, one BEL1-like homeobox gene, *BEL1* (Tai6. 36,202), was strongly upregulated (50-fold increase in expression) in storage roots at the early stage compared with fibrous roots, which indicated that this gene might be a positive regulator during storage root formation, whereas the opposite expression profile was obtained for *BEL5*, which indicated that this gene might act as a negative regulator (Fig. 7b). However, further research is necessary to determine whether the expression of these genes is correlated with the regulation of storage root development in sweet potato. In addition, previous studies have shown that *Arabidopsis* BEL1 homeodomain protein can selectively heterodimerize with specific KNAT homeodomain proteins through a MEINOX domain that is conserved between plants and animals [38]. The MEINOX domain, which serves as an interaction domain for developmental regulators, might also be conserved in sweet potatoes.

MADS-box transcription factors are reportedly essential regulators of storage root development. Kim et al. [16, 17] reported five MADS-box genes, namely, *IbMADS3*, *IbMADS4*, *IbMADS79*, *IbAGL17* and *IbAGL20*, that are highly expressed in storage roots. The heterologous overexpression of the sweet potato AGL17 MADS-box gene *IbMADS1* in potato causes the fibrous roots of potato to exhibit tuber morphogenesis [18]. Noh et al. [11] reported that *SRD1* regulates the formation and bulking of sweet potato storage roots by promoting the initiation of cambium and metaxylem to regulate the storage root formation in an auxin-dependent manner. Our transcriptome data showed that 74 MADS-box genes were differentially expressed in fibrous roots and storage roots (Additional file 2: Table S1). The genes in cluster I, which included *AGL15* (Tai6.15221), were gradually upregulated starting at the early stage of storage root development (Additional file 3: Table S2). These results indicated that homeobox and MADS-box genes might play critical roles in the formation and development of storage roots by regulating the formation of meristems and the content of endogenous hormone contents.

Additionally, both our transcriptome and proteome data revealed that the NAC, bZIP and MYB transcription factors were also differentially expressed in fibrous roots compared with storage roots (Additional file 7: Table S4 and Fig. 7). Previous high-throughput sequencing data showed that two NAC genes are downregulation in storage roots [19]. Both our transcriptome and proteome analyese suggested that *NAC1* (Tai6.49584) showed a gradual downregulation in storage roots compared with fibrous roots (Fig. 7 and Additional file 7: Table S4), which indicated that *NAC1* might act as an inhibitor in the development of storage roots. Moreover, our findings revealed that NAC transcription factors were related to starch synthesis. For example, the overexpression of *ZmNAC36* induces the expression of a considerable number of starch synthetic genes [39]. Additionally, Xiao et al. [40] reported that a maize MYB transcription factor *ZmMYB14* serves as a key regulator in starch biosynthesis by directly binding to promoters of starch synthesis-related genes. Furthermore, the bZIP transcription factor AtbZIP63 affects the starch degradation and glucose signals in *Arabidopsis thaliana* [41]. Together, these results indicated that bZIP and MYB transcription factors might be related to starch biosynthesis in sweet potato storage roots. Nevertheless, these transcription factors deserve further investigation as potentially significant regulators in the development of storage roots.

## Conclusions

A combination of transcriptome and proteome analyses, as well as anatomical and physiological observations, was performed in the present study. Multiple storage root development-associated events, including cambium development, starch accumulation, and changes in endogenous hormones and several key candidate regulators, were revealed. The identification of specific genes that regulate storage root formation in sweet potato remains an important but challenging goal in developmental biology research. Gene engineering has been shown to have great potential for improving the yield and quality of this crop. Although many additional studies are needed to elucidate their functions, our present results provide a series of candidate genes and proteins that could be applied in the breeding of high-yield sweet potato and improve our understanding of the molecular mechanism underlying the regulatory networks of storage root development.

## Methods

### Plant materials

Sweet potato (*Ipomoea batatas* (L.) Xushu22) tuberous roots were obtained from the Sweet Potato Research Institute, Xuzhou Academy of Agricultural Sciences, National Sweet Potato Industry System, China. The tuberous roots were planted in plastic pots for approximately one month, ans shoots with five to six functional leaves were then cut from the tuberous roots and transplanted in a greenhouse with a temperature of 18~28 °C and a long-day photoperiod (16 h of light/8 h of dark). Fibrous roots (F; root diameters of approximately 1 mm) and storage roots at four stages (D1, D3, D5 and D10; root diameters of 1 cm, 3 cm, 5 cm and 10 cm respectively) from the sweet potato plants were collected at 15, 30, 60, 90 and 120 days after transplantation, respectively, to cover the entire root development processes. These materials were divided into two parts: one part was immediately frozen in liquid nitrogen and subsequently stored at − 80 °C for hormone, RNA and protein isolation, and the other part was used immediately for anatomical observations.

### Anatomical observations

For anatomical observations, fresh root tissues were sectioned and immediately immersed in fixative. The sectioned tissues were then dehydrated and subjected to paraffin infiltration as follows: 75% alcohol, 4 h; 85% alcohol, 2 h; 90% alcohol, 2 h; 95% alcohol, 1 h; 100% alcohol I, 0.5 h; 100% alcohol II, 0.5 h; alcohol and xylene mix, 5 to 10 min; xylene I, 5 to 10 min; xylene II, 5 to 10 min; paraffin (65 °C) I, 1 h; paraffin (65 °C) II, 1 h; and paraffin (65 °C) III, 1 h. Subsequently, these tissue samples were processed in melted paraffin in cassettes and frozen at − 20 °C until the paraffin had completely solidified. The paraffin blocks were then removed and appropriately trimmed. Sections (4 μm) were cut using a microtome, picked up with a paint brush and placed on the surface of a deionized water bath at 40 °C. The sections were floated onto histological slides, and the slides were dried in an oven (60 °C) and then stored at room temperature. Finally, the tissues were viewed with a tissue scanner (3D HISTECH, Pannoramic MIDI).

### Transcriptome analysis

For transcriptome analysis, the tissues of fibrous roots (F) and storage roots at different stages (D1, D3, D5 and D10) were collected from sweet potato plants, and the total RNAs were extracted. The RNA-seq sequencing and assembly were performed by Novogene Co., LTD (Beijing, China). A total amount of 3 μg of RNA from each sample was used as the input material. Clean reads were obtained by removing reads containing an adapter, reads containing ploy-N and low-quality reads from the raw data. The clean reads were then aligned with the sweet potato genome (http://public-genomes-ngs.molgen.mpg.de/cgi-bin/hgGateway?hgsid=9052&clade=plant&org=Ipomoea+batatas&db=ipoBat4). FeatureCounts v 1.5.0-p3 was used to count the read numbers mapped to each gene, and the FPKM of each gene was then calculated based on the length of the gene and the read count mapped to the gene. Genes with an adjusted *P*-value < 0.05 obtained by DESeq2 were considered DEGs. Gene Ontology (GO) enrichment analysis of the DEGs was implemented using the cluster Profiler R package, and the gene length bias was corrected during this process [42]. KOBAS software was used to test the statistical enrichment of the DEGs in Kyoto Encyclopedia of Genes and Genomes (KEGG) pathways [43]. To obtain more information about the DEGs, the DEGs were annotated using seven databases: NR (NCBI nonredundant protein), NT (NCBI Nucleotide Sequences), Gene Ontology (GO), KO (KO, KEGG Orthology), KOG (Eukaryotic Orthologous Groups), Pfam (Protein Family Database) and Swiss-Prot (a manually annotated and reviewed protein sequence database). All the DEGs were subjected to hierarchical clustering analysis using the average linkage method [44].

### Proteome analysis

The iTRAQ analysis of the proteome was performed by Novogene Co., LTD (Beijing, China). The total protein from the fibrous roots (F) and storage roots at different stages (D1, D3, D5 and D10) was extracted. The protein concentration was determined using the Bradford method with BSA as the standard, and the protein purity was examined by 10% sodium dodecyl sulfate-polyacrylamide gel electrophoresis (SDS-PAGE). After trypsin digestion, the peptides were dried by vacuum centrifugation, and the desalted peptides were labeled with iTRAQ reagents (iTRAQ® Reagent-8PLEX Multiplex Kit, Sigma) following the manufacturer’s instructions (AB Sciex, Foster City, CA, USA). An ~ 600-μg iTRAQ-labeled peptide mix was fractionated using a C18 column (waters BEH C18 4.6 × 250 mm, 5 μm) on a Rigol L3000 HPLC operated at 1 ml/min. The resulting spectra from each fraction were searched separately against the sweet potato genome (http://public-genomes-ngs.molgen.mpg.de/SweetPotato/) database using the Proteome Discoverer 2.2 search engine (PD 2.2, Thermo). The protein quantitation results were statistically analyzed by the Mann-Whitney test, and the significant ratios, defined by *p* < 0.05 and |log2(fold change)| ≥1, were used to screen the differentially expressed proteins (DEPs). GO and InterPro (IPR) analyses were conducted using the InterProScan-5 program against nonredundant protein databases (including Pfam, PRINTS, ProDom, SMART, ProSiteProfiles, and PANTHER), and the COG (Clusters of Orthologous Groups) and KEGG databases were used to analyze the protein families and pathways.

### Real-time quantitative PCR validation

The total RNAs from sweet potato fibrous roots (F) and storage roots at four stages (D1, D3, D5 and D10) were isolated and reverse-transcribed using the PrimeScript™ RT reagent kit with gDNA Eraser (Takara) according to the user’s manual. The qPCR and data analyses were performed as described by Cai et al. [45]. Fourteen DEGs, including three starch biosynthesis-related genes, five plant hormone biosynthesis-related genes and six transcription factors from the RNA-Seq and proteome, were validated, and the primers used for this validation are listed in Additional file 15: Table S8. Sweet potato *ARF* (ADP-ribosylation factor) was used as the reference gene for the normalization of gene expression [46].

### Analysis of starch content

The total starch content in 0.1 g of sweet potato root material was analyzed by spectrophotometry according to the instruction manual provided with the Starch Content Assay Kit (Solarbio Life Science, Beijing).

### Quantification of endogenous plant hormones

The contents of endogenous plant hormones (IAA, GAs, JA, CTK and ABA) in 0.1 g of sweet potato root material were determined by ELISA according to the instructions provided with the Plant IAA ELISA Kit, Plant GA ELISA Kit, Plant JA ELISA Kit, Plant CTK ELISA Kit and Plant ABA ELISA Kit (MiBio, Shanghai), respectively.

## Additional files

Additional file 1: **Figure S1.** The number of genes annotated in seven databases of transcriptome. (PDF 115 kb)

Additional file 2: **Table S1.** Gene description and expression in transcriptome. (XLSX 75423 kb)

Additional file 3: **Table S2.** Gene information in four sub-clusters respectively. (XLS 8363 kb)

Additional file 4: **Figure S2.** GO analysis of four clusters in transcriptome. (PDF 219 kb)

Additional file 5: **Table S3**. The detailed information of unigenes in each GO functional category of transcriptome analysis. (XLS 72 kb)

Additional file 6: **Figure S3.** The number of proteins annotated in four databases of proteome. (PDF 164 kb)

Additional file 7: **Table S4.** Correlation between transcriptome and proteome. (XLSX 1344 kb)

Additional file 8: **Figure S4.** GO analysis of six clusters in proteome. (PDF 267 kb)

Additional file 9: **Table S5.** The detailed information of each GO functional category of proteomic analysis. (XLS 1085 kb)

Additional file 10: **Figure S5.** The number of transcripts and proteins between storage roots and fibrous roots. (PDF 170 kb)

Additional file 11: **Table S6.** Candidate genes in storage roots vs fibrous roots of sweet potato in both transcriptome and proteome. (DOCX 19 kb)

Additional file 12: **Figure S6.** mRNA (A) and protein (B) expression analysis of related genes detected in fibrous roots (F) and storage roots of D1, D3, D5 and D10 stages. (PDF 435 kb)

Additional file 13: **Table S7.** Different expressed transcription factors in transcriptome analysis. (XLS 6892 kb)

Additional file 14: **Figure S7.** Multiple alignment of BEL1 and BEL5 proteins. (PDF 198 kb)

Additional file 15: **Table S8.** Primer pairs used in real-time quantitative RT-PCR. (XLS 35 kb)

## Abbreviations

ABA: Abscisic acid
COG: Cluster of orthologous groups of proteins
CTKs: Cytokinin
D1: Storage roots with about 1 cm diameters
D10: Storage roots with about 10 cm diameters
D3: Storage roots with about 3 cm diameters
D5: Storage roots with about 5 cm diameters
DEGs: Differentially expressed genes
DEPs: Differentially expressed proteins
F: Fibrous roots
GAs: Gibberellin
GLGB: Starch-branching enzyme
GLGL: ADP-glucose pyrophosphorylase
GO: Gene ontology
IAA: Indole acetic acid
IPR: Iproclass protein database
iTRAQ: Isobaric tags for relative and absolute quantitation
JA: Jasmonic acid
KEGG: Kyoto encyclopedia of genes and genomes
KO: KEGG orthology
KOG: Eukaryotic orthologous groups
LBD4: LOB domain-containing protein 4
NR: NCBI non-redundant protein
NT: NCBI nucleotide sequences
Pfam: Protein family database
SSY: Starch synthase
TMO6: TARGET OF MONOPTEROS 6
WOX4: WUSCHEL HOMEOBOX RELATED 4
