# Supplementary material for: RNA-Seq and iTRAQ reveal multiple pathways involved in storage root formation and development in sweet potato (Ipomoea batatas L.)
Source: BMC Plant Biol. 2019 Apr 11;19:136. doi: 10.1186/s12870-019-1731-0 (PMC6458706; doi:10.1186/s12870-019-1731-0)
Supplement: Supplementary file 1 — Figure S1. The number of genes annotated in seven databases of transcriptome. (PDF 115 kb) [file 12870_2019_1731_MOESM1_ESM.pdf]

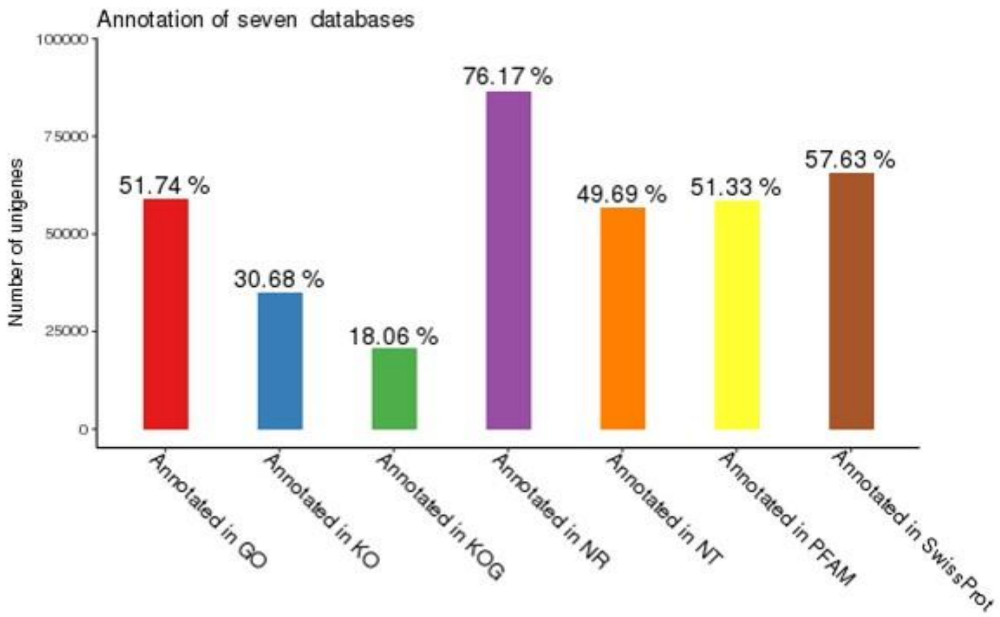


**Fig. S1.** The number of genes annoated in seven databases of transcriptome. NR, NCBI Non-redundant Protein; NT, NCBI Nucleotide Sequences; GO, Gene Ontology; KO, KEGG Orthology; KOG, Eukaryotic Orthologous Groups; Pfam, Protein Family Database; Swissprot, A manually annotated and reviewed protein sequence database.
