# Supplementary material for: RNA-Seq and iTRAQ reveal multiple pathways involved in storage root formation and development in sweet potato (Ipomoea batatas L.)
Source: BMC Plant Biol. 2019 Apr 11;19:136. doi: 10.1186/s12870-019-1731-0 (PMC6458706; doi:10.1186/s12870-019-1731-0)
Supplement: Supplementary file 4 — Figure S2. GO analysis of four clusters in transcriptome. (PDF 219 kb) [file 12870_2019_1731_MOESM4_ESM.pdf]

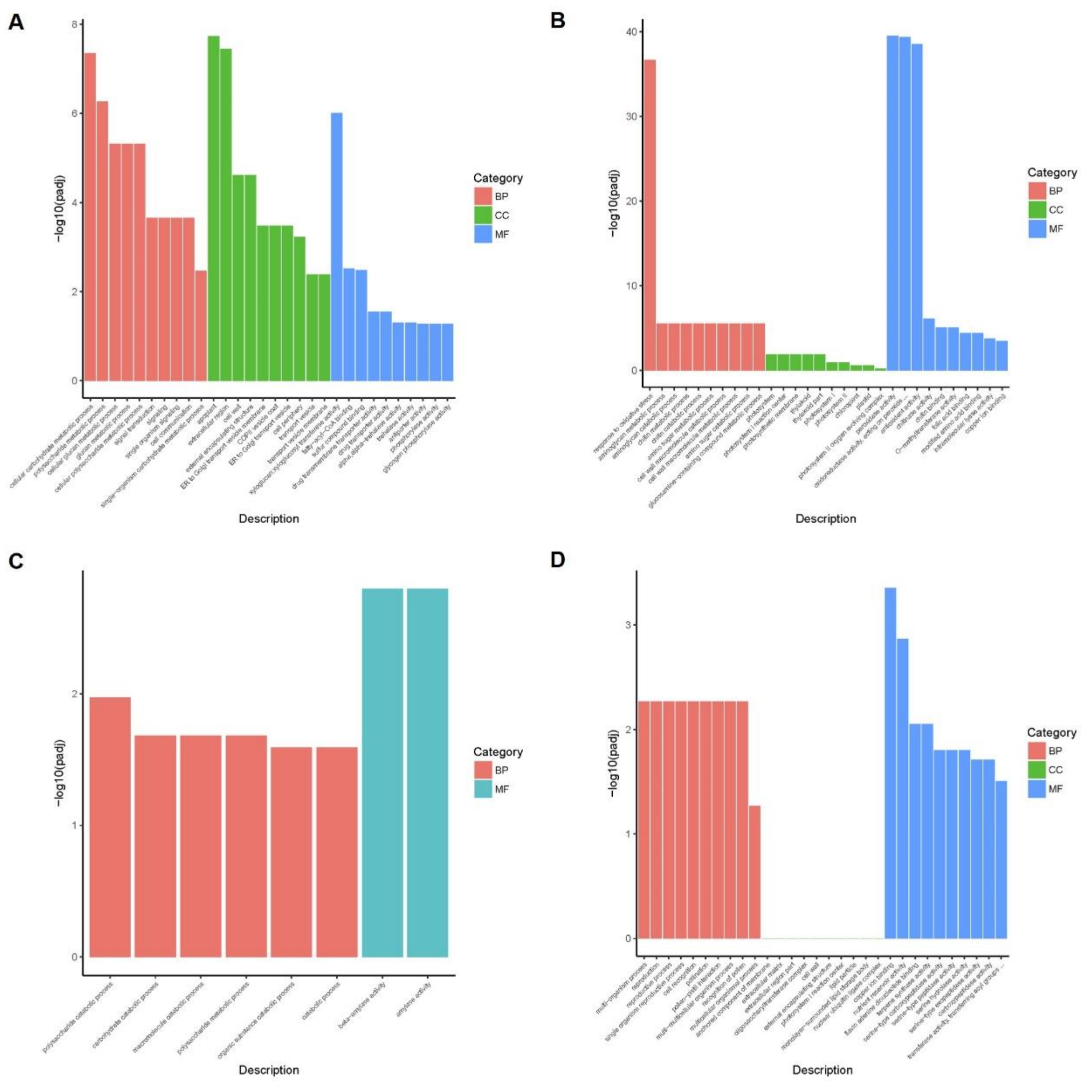

Fig. S2. GO analysis of four clusters in transcriptome. (A) GO analysis of cluster I in transcriptome. (B) GO analysis of cluster II in transcriptome. (C) GO analysis of cluster III in transcriptome. (D) GO analysis of cluster IV in transcriptome.
