# Supplementary material for: RNA-Seq and iTRAQ reveal multiple pathways involved in storage root formation and development in sweet potato (Ipomoea batatas L.)
Source: BMC Plant Biol. 2019 Apr 11;19:136. doi: 10.1186/s12870-019-1731-0 (PMC6458706; doi:10.1186/s12870-019-1731-0)
Supplement: Supplementary file 6 — Figure S3. The number of proteins annotated in four databases of proteome. (PDF 164 kb) [file 12870_2019_1731_MOESM6_ESM.pdf]

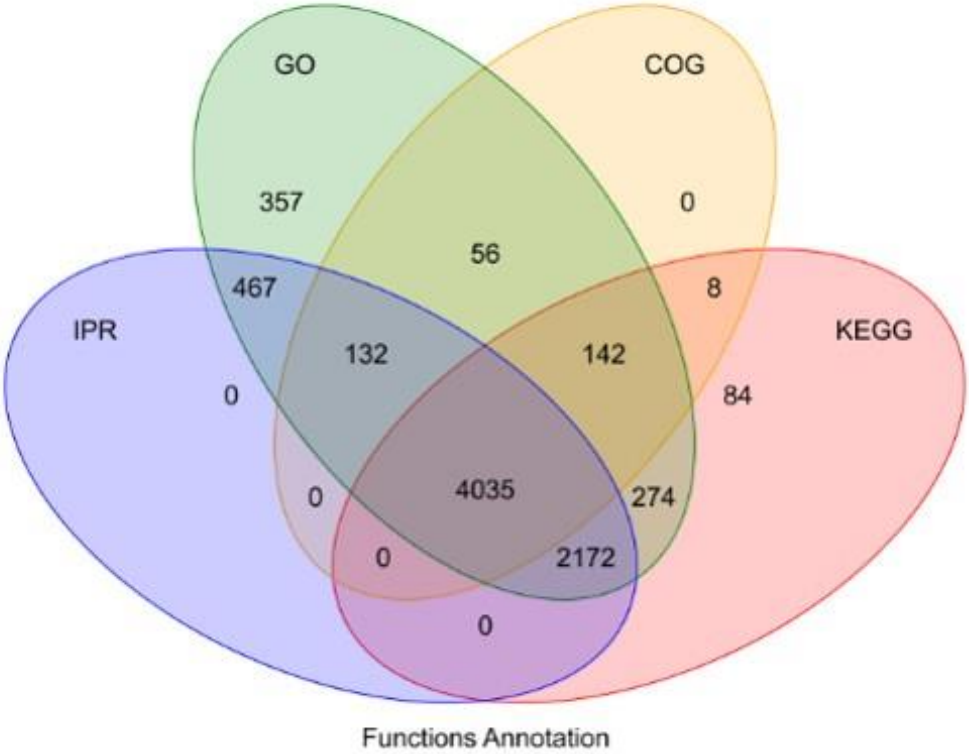

Fig. S3. The number of proteins annoated in four databases of proteome. GO, Gene Ontology; COG, Cluster of Orthologous Groups of Proteins; IPR, iProclass Protein Database; KEGG, Kyoto Encyclopedia of Genes and Genomes.
