# Supplementary material for: RNA-Seq and iTRAQ reveal multiple pathways involved in storage root formation and development in sweet potato (Ipomoea batatas L.)
Source: BMC Plant Biol. 2019 Apr 11;19:136. doi: 10.1186/s12870-019-1731-0 (PMC6458706; doi:10.1186/s12870-019-1731-0)
Supplement: Supplementary file 8 — Figure S4. GO analysis of six clusters in proteome. (PDF 267 kb) [file 12870_2019_1731_MOESM8_ESM.pdf]

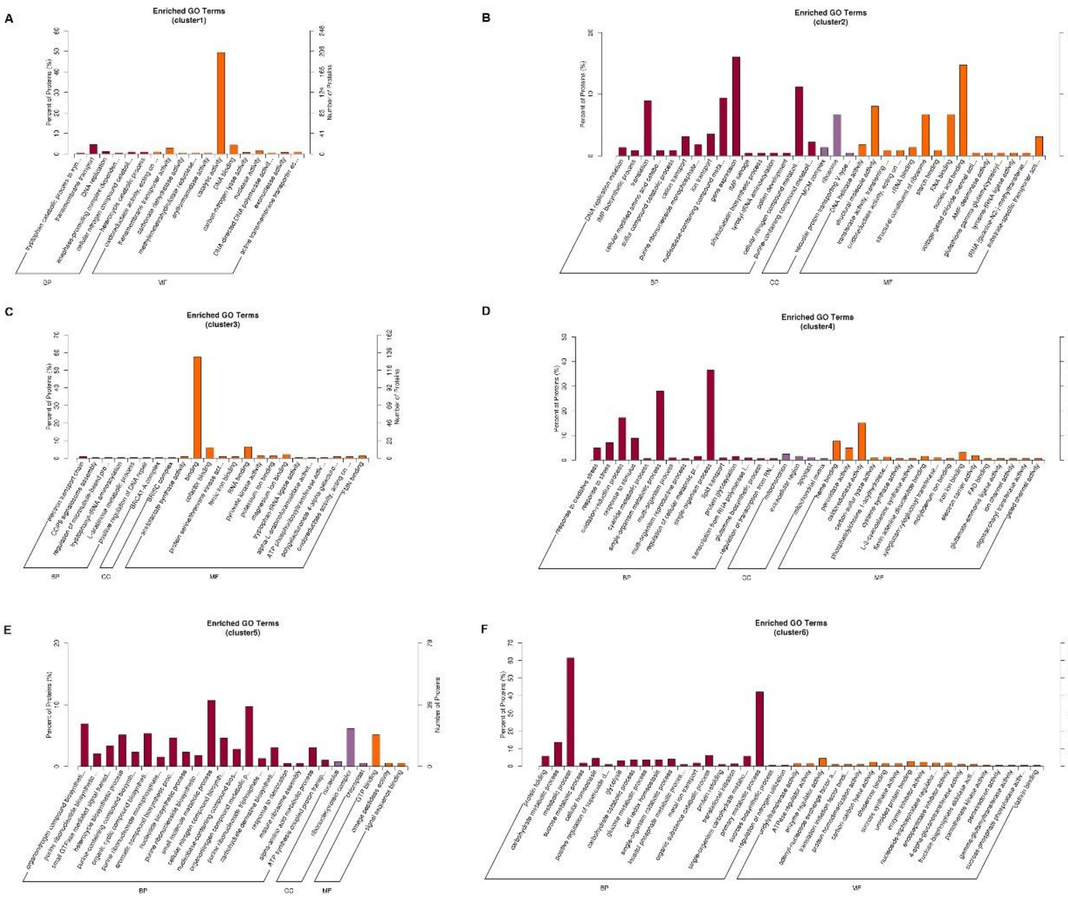

Fig. S4. GO analysis of six clusters in proteome. (A) GO analysis of cluster 1 in transcriptome. (B) GO analysis of cluster 2 in transcriptome. (C) GO analysis of cluster 3 in transcriptome. (D) GO analysis of cluster 4 in transcriptome. (E) GO analysis of cluster 5 in transcriptome. (F) GO analysis of cluster 6 in transcriptome.
