# Supplementary material for: RNA-Seq and iTRAQ reveal multiple pathways involved in storage root formation and development in sweet potato (Ipomoea batatas L.)
Source: BMC Plant Biol. 2019 Apr 11;19:136. doi: 10.1186/s12870-019-1731-0 (PMC6458706; doi:10.1186/s12870-019-1731-0)
Supplement: Supplementary file 10 — Figure S5. The number of transcripts and proteins between storage roots and fibrous roots. (PDF 170 kb) [file 12870_2019_1731_MOESM10_ESM.pdf]

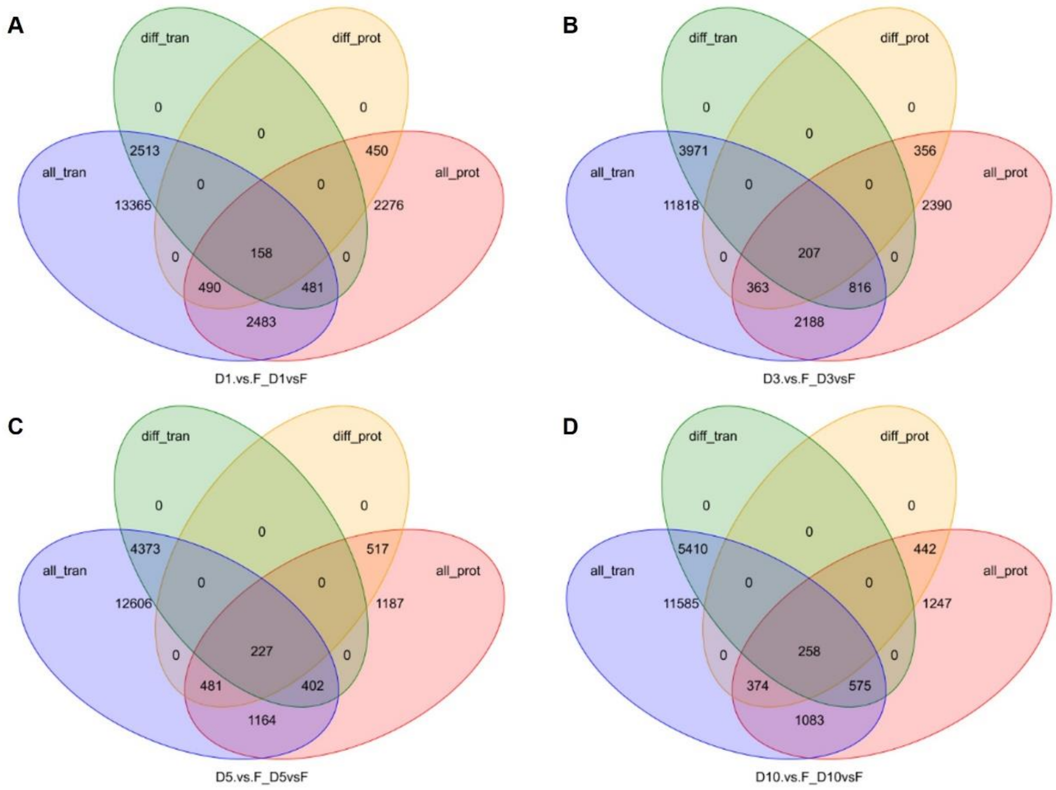

Fig. S5. The number of transcripts and proteins between storage roots and fibrous roots. (A) The number of transcripts and proteins between D1-stage of storage roots and fibrous roots. (B) The number of transcripts and proteins between D3-stage of storage roots and fibrous roots. (C) The number of transcripts and proteins between D5-stage of storage roots and fibrous roots. (D) The number of transcripts and proteins between D10-stage of storage roots and fibrous roots.
