# Supplementary material for: RNA-Seq and iTRAQ reveal multiple pathways involved in storage root formation and development in sweet potato (Ipomoea batatas L.)
Source: BMC Plant Biol. 2019 Apr 11;19:136. doi: 10.1186/s12870-019-1731-0 (PMC6458706; doi:10.1186/s12870-019-1731-0)
Supplement: Supplementary file 11 — Table S6. Candidate genes in storage roots vs fibrous roots of sweet potato in both transcriptome and proteome. (DOCX 19 kb) [file 12870_2019_1731_MOESM11_ESM.docx]

**Table S6.** Candidate genes in storage roots vs fibrous roots of sweet potato in both transcriptome and proteome

|  | **Gene Name** | **Target Description** | **NO. all^a^** | **Transcriptome Analysis** | | **Proteome**  **Analysis** | |
| --- | --- | --- | --- | --- | --- | --- | --- |
|  |  |  |  | **NO. up^b^** | **NO. down^c^** | **NO. up^d^** | **NO. down^e^** |
| **Starch Biosynthetic genes** | *UGPA* | UDP-glucose pyrophosphoryloase | 1 | 1 | 0 | 1 | 0 |
|  | *GLGL* | ADP-glucose pyrophosphoryloase | 1 | 1 | 0 | 0 | 1 |
|  | *SSY* | Starch synthase | 5 | 5 | 0 | 5 | 0 |
|  | *GLGB* | Starch branching enzymes | 3 | 3 | 0 | 3 | 0 |
| **Hormone Biosynthetic genes** | *NIT4* | Nitrile aminohydrolase 4 | 1 | 1 | 0 | 0 | 1 |
|  | *APRT* | Adenine hosphoribosyl transferase | 1 | 1 | 0 | 1 | 0 |
|  | *GA3ox4* | Gibberellin 3-β-dioxygenase 4 | 2 | 0 | 2 | 0 | 2 |
|  | *OPR3* | OPDA reductase 3 | 2 | 1 | 1 | 1 | 1 |
|  | *AOS* | Allene oxide synthase | 2 | 1 | 1 | 0 | 2 |
|  | *ZEP* | Zeaxanthin epoxidase | 3 | 1 | 2 | 0 | 3 |
|  | *SDR* | Short-chain alcohol dehydrogenasel reductase | 1 | 1 | 0 | 0 | 1 |
|  | *AAO* | ABA-aldehyde oxidase | 1 | 1 | 0 | 1 | 0 |
| **Transcription Factor** | *Homeobox* | homeodomain transcription factors | 15 | 7 | 8 | 10 | 5 |
|  | *MYB* | MYB transcription factors | 8 | 7 | 1 | 2 | 6 |
|  | *bZIP* | basic region/leucine zipper transcription factors | 15 | 10 | 5 | 9 | 6 |
|  | *NAC* | NAM/ATAF/CUC transcription factors | 6 | 5 | 1 | 4 | 2 |

a.Number of differentially expressed genes/proteins identified in storage roots compared with fibrous roots of sweet potato in both transcriptome and proteome. b. Number of up-regulated genes identified in storage roots compared with fibrous roots of sweet potato in transcriptome. c. Number of down-regulated genes identified in storage roots compared with fibrous roots of sweet potato in transcriptome. d. Number of up-regulated genes identified in storage roots compared with fibrous roots of sweet potato in proteome. e. Number of down-regulated genes identified in storage roots compared with fibrous roots of sweet potato in proteome.
