# Supplementary material for: RNA-Seq and iTRAQ reveal multiple pathways involved in storage root formation and development in sweet potato (Ipomoea batatas L.)
Source: BMC Plant Biol. 2019 Apr 11;19:136. doi: 10.1186/s12870-019-1731-0 (PMC6458706; doi:10.1186/s12870-019-1731-0)
Supplement: Supplementary file 12 — Figure S6. mRNA (A) and protein (B) expression analysis of related genes detected in fibrous roots (F) and storage roots of D1, D3, D5 and D10 stages. (PDF 435 kb) [file 12870_2019_1731_MOESM12_ESM.pdf]

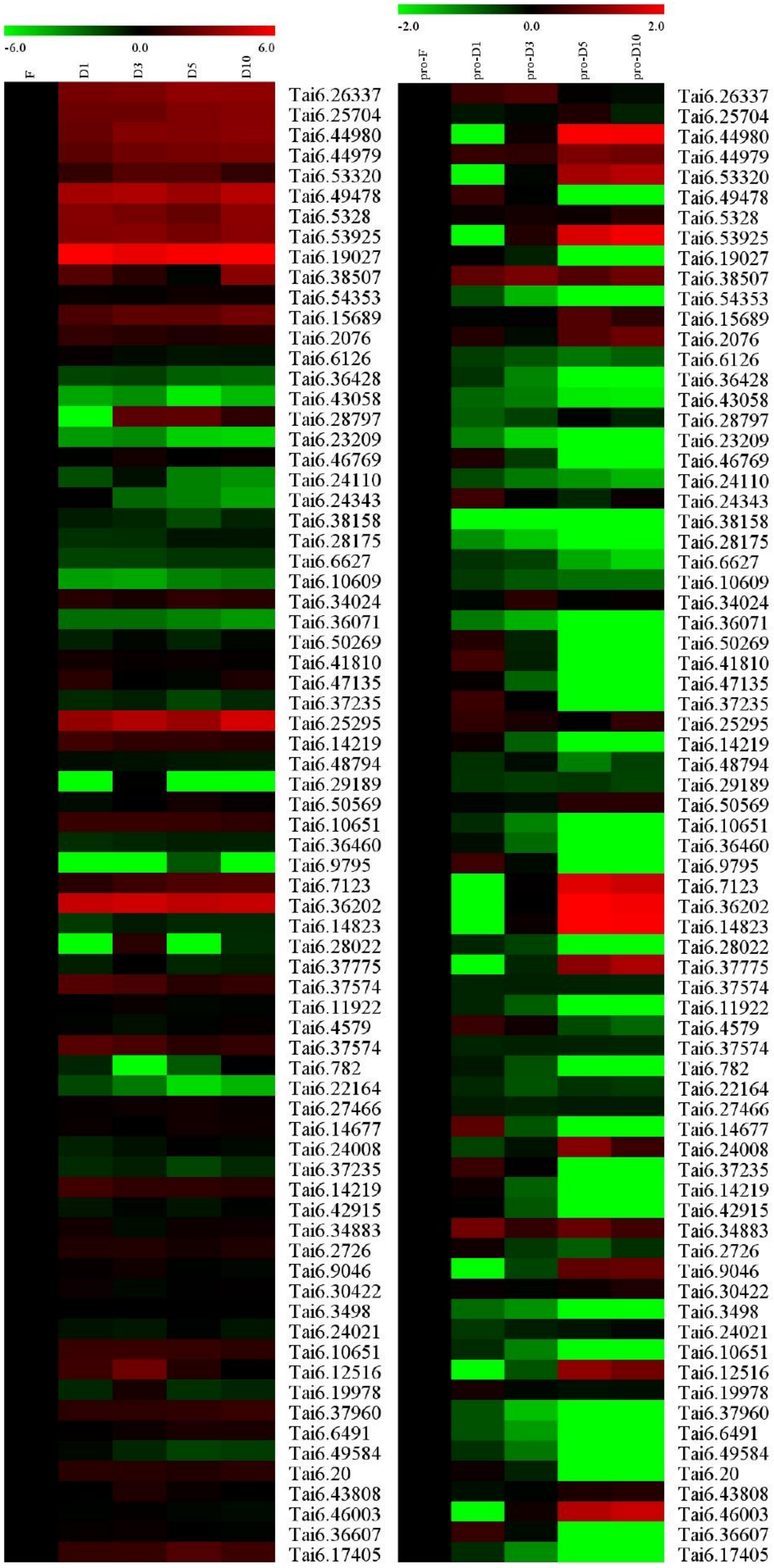

Fig. S6. mRNA (A) and protein (B) expression analysis of related genes detected in fibrous roots (F) and storage roots of D1, D3, D5 and D10 stages. Log_2-transformed fold-change data were used for producing the heatmaps by MeV4.9. The expression levels of fibrous roots (F) were set to 1. The color scales at top indicate the relative expression. Red and green shading indicates the relative high or low expression levels, respectively.
