# Supplementary material for: RNA-Seq and iTRAQ reveal multiple pathways involved in storage root formation and development in sweet potato (Ipomoea batatas L.)
Source: BMC Plant Biol. 2019 Apr 11;19:136. doi: 10.1186/s12870-019-1731-0 (PMC6458706; doi:10.1186/s12870-019-1731-0)
Supplement: Supplementary file 14 — Figure S7. Multiple alignment of BEL1 and BEL5 proteins. (PDF 198 kb) [file 12870_2019_1731_MOESM14_ESM.pdf]

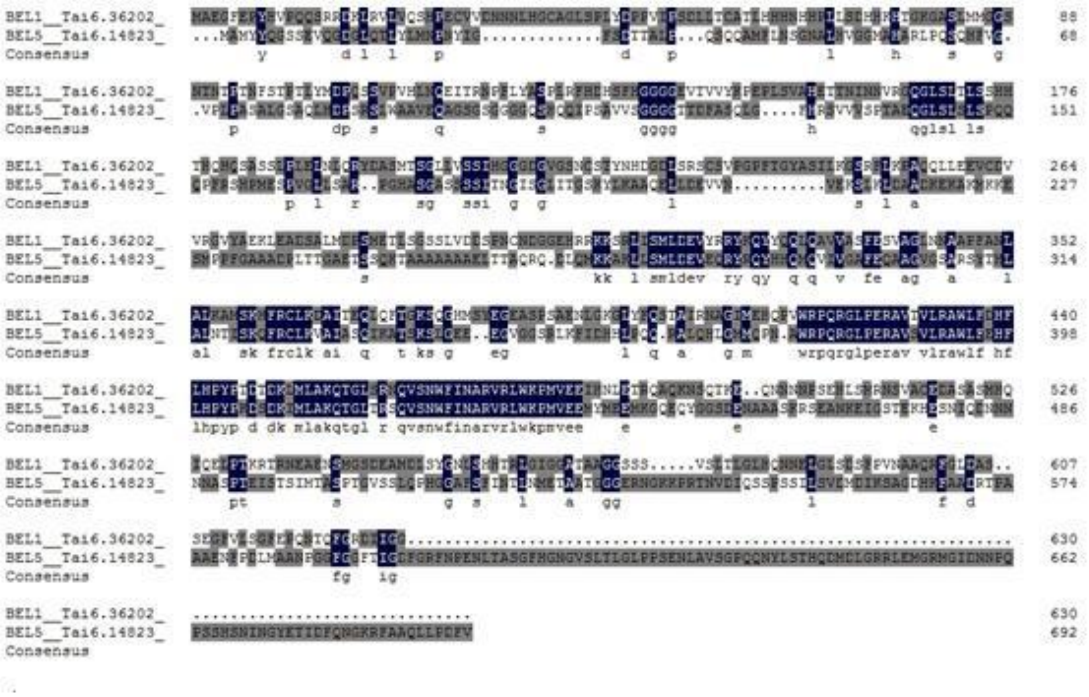

Fig. S7. Multiple alignment of BEL1 and BEL5 proteins. Identical amino acids are highlighted in blue and similar amino acids in gray.
